# Speech Cues Contribute to Audiovisual Spatial Integration

**DOI:** 10.1371/journal.pone.0024016

**Published:** 2011-08-31

**Authors:** Christopher W. Bishop, Lee M. Miller

**Affiliations:** 1 Center for Mind and Brain, University of California Davis, Davis, California, United States of America; 2 Department of Neurobiology, Physiology and Behavior, University of California Davis, Davis, California, United States of America; University of Regensburg, Germany

## Abstract

Speech is the most important form of human communication but ambient sounds and competing talkers often degrade its acoustics. Fortunately the brain can use visual information, especially its highly precise spatial information, to improve speech comprehension in noisy environments. Previous studies have demonstrated that audiovisual integration depends strongly on spatiotemporal factors. However, some integrative phenomena such as McGurk interference persist even with gross spatial disparities, suggesting that spatial alignment is not necessary for robust integration of audiovisual place-of-articulation cues. It is therefore unclear how speech-cues interact with audiovisual spatial integration mechanisms. Here, we combine two well established psychophysical phenomena, the McGurk effect and the ventriloquist's illusion, to explore this dependency. Our results demonstrate that conflicting spatial cues may not interfere with audiovisual integration of speech, but conflicting speech-cues can impede integration in space. This suggests a direct but asymmetrical influence between ventral ‘what’ and dorsal ‘where’ pathways.

## Introduction

Our brains continually integrate information from multiple sensory systems to improve perception [1], [2], [3], [4], [5]. For instance, watching a speaker's lip movements significantly enhances speech intelligibility [3], [4], especially when speech is degraded by reverberations or competing talkers (e.g. at a cocktail party [6]). Furthermore, the brain can use visual information to refine unreliable auditory spatial estimates [7], [8], [9], [10]. Previous research has clearly demonstrated the general importance of low-level stimulus attributes, such as spatial and temporal coincidence, in these integrative processes [11], [12], [13], [14], [15]. Specifically, the likelihood of integration decreases with increasing spatiotemporal disparity. Although space and time are widely accepted as important factors in integration, not all integrative processes require strict spatial alignment. For instance, the McGurk illusion [16] (e.g. when subjects are presented with an auditory /aba/ and visual /aga/ they typically report hearing /ada/) persists even with large spatial differences [17]. Thus, it seems that spatial information does not influence higher order processing of speech stimuli under all circumstances. However we do not know, conversely, whether speech cues influence basic multisensory processing of space. In this study, we directly test the hypothesis that phonetically incongruent audiovisual speech affects the integration of spatial information by measuring the effect of phonetically congruent and incongruent stimuli (i.e. McGurk pairs [16]) on the ventriloquist's illusion [18].

The ventriloquist's illusion provides a powerful assay of audiovisual spatial integration. Subjects often experience the illusion when presented with spatially disparate audiovisual stimuli (e.g. [15]). Typically, the perceived location of the sound is captured by the visual cues; however for a range of spatial disparities, this capture may succeed on some trials and fail on others, even with physically identical stimuli. The illusion can thus be harnessed as a direct index for ongoing spatial integration of sight and sound in the absence of stimulus confounds. Although studies have demonstrated that spatial integration, as measured by the ventriloquist's illusion, is susceptible to high order cognitive variables such as the “cognitive compellingness” of the stimulus set used [19], it is not clear how relatively high-order phonetic cues specifically affect these integrative processes.

In this study, we explored the relationship between audiovisual integration of spatial and phonetic cues. To do this, we used well described audiovisual illusions as metrics for integration in each domain: the ventriloquist's illusion in space [18] and McGurk interference for speech related cues [16]. Furthermore, to control and adequately sample acoustic space, we simulated it with subject specific head related transfer functions (HRTFs). We hypothesized that integration of speech cues, such as the place of articulation important for McGurk interference, would operate independently of audiovisual spatial relationships; in contrast, we hypothesized that speech cues would have a significant impact on audiovisual spatial integration.

## Methods

### Subjects and Ethics Statement

Fifteen healthy subjects (10 women, ages 19–26 yrs., mean 22 yrs.; 5 participated in an HRTF Validation Experiment, see below for details) gave written consent according to procedures approved by the University of California, Davis Institutional Review Board (UCD IRB) and were paid for their participation in the UCD IRB approved experiment described here. Participants learned English as an infant, had self-reported good hearing, and normal or corrected-to-normal vision. Four subjects (3 female) were excused from the study prior to data collection due to technical difficulties in estimating their head related transfer functions. Consequently, the reported findings include the remaining 11 (7 female) subjects.

### Stimuli

Four consonants (/b/, /g/, /k/, /p/) were paired with /a/ to produce four vowel-consonant-vowel (VCV) speech tokens. These VCVs were spoken by a female actress with voice training and recorded using a digital camcorder and remote microphone. Video was acquired at 29.97 Hz and audio at 48 kHz. Videos were subsequently resampled at 60 Hz, converted to black and white, and luminance-normalized using in-house scripts. A single instance of each VCV was used in the experiment. These tokens were selected to maximize the temporal alignment (<5 ms offset at speech and consonant onset, well within the duration of a single frame of the 60 Hz video) and match the pitch and timbre of phonetically congruent and incongruent pairs. Sounds were presented dichotically at ∼70 dB through ER-4B headphones (www.etymotic.com). All auditory stimuli were digitally filtered to compensate for the specific frequency response of the sound playback and recording equipment. White noise was presented diotically at approximately 54 dB to mask any transients introduced during speech filtering (see below). This resulted in an approximate speech-to-noise ratio of +16 dB.

### Head related transfer function (HRTF) estimation

Individualized head related transfer functions (HRTFs) were used to create a virtual acoustic environment for each subject. Importantly, these were deliberately acquired in a reverberant room to improve sound externalization and prevent sounds from appearing “in the head”, a problem that has limited the use of HRTFs in studies of spatial hearing [20]. Thus, each subject's location-specific transfer functions contained both aspects of the subject's HRTF and the room impulse response. Although describing these estimates as a pure HRTF is technically inaccurate, we use this notation rather than its proper notation (binaural room impulse response or BRIR) for clarity of presentation.

HRTFs were estimated by presenting 3 s of white noise from a Tannoy Precision 6D (www.tannoy.com) free-field monitor located approximately 2 m from the subject and recording the signal at the entrance of the subject's ear canals using AuSIM inner ear microphones (www.ausim3d.com) at a rate of 96 kHz. Subject- and location-specific transfer functions were calculated in MATLAB (www.mathworks.com) by dividing the Fast Fourier Transform (FFT) of the recording in each ear by the FFT of the original white noise sample at each location. The procedure was repeated at locations ranging from −36° (left of midline) +36° (right of midline) in 6° increments. Auditory speech stimuli were positioned in virtual space by filtering VCVs with a truncated (80 ms) time representation of the HRTF called the head related impulse response (HRIR). This resulted in robust externalization, minimized unwanted anomalies (e.g. “front-back confusion”), and maintained the discrete spatial nature of the signal (see Figure 2).

### Head related transfer function (HRTF) validation

HRTFs for ten healthy individuals (6 female, mean age 24, 5 participated in main experiment) were acquired as described above and the accuracy of perceived auditory space was assessed using a pointing task. Subjects sat in a comfortable, rotatable chair equipped with a custom headrest and laser pointer suspended directly above the subject's head. The subject was instructed to fixate on a marking located straight ahead (0°) during sound presentation. A single vowel-consonant-vowel (VCV) was then presented at one of thirteen virtual locations ranging from −36° to +36° in random order. Following sound offset, the subject oriented her body towards the perceived azimuthal location by rotating in the chair. The location of the laser pointer was recorded by the experimenter and converted to azimuthal angle in MATLAB. The subject returned to the starting position and the procedure was repeated for a total of twenty-six trials (two per location).

### Task Procedures

**Figure 1:**
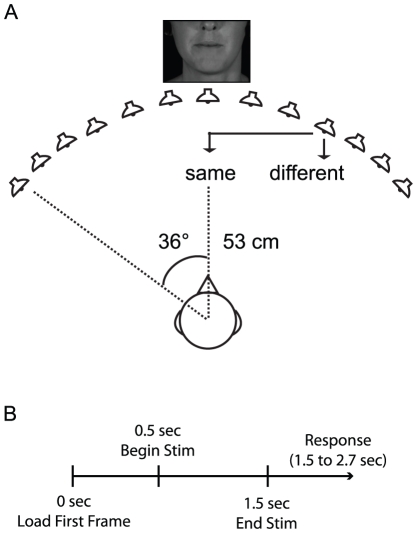
Stimulus setup and trial structure. (A) Subjects sat approximately 53 cm from a video of the speaker's face. Sounds were positioned at 13 different locations ranging from −36° to +36° in 6° increments. Subjects reported whether they perceived the presented sound to originate from the *Same-* or *Different-Location* as the speaker's mouth. Arrows point toward the perceived location and the corresponding response by the subject. (B) Trial structure. The first frame of each video was loaded 500 ms prior to starting the full video. Subjects were allowed 1.2 s to respond.

Subjects sat in a comfortable chair in a dimly lit room and placed their chin in a chinrest to prevent head movements. Visual stimuli were presented on a Dell FPW2407 (www.dell.com) situated approximately 53 cm from the subject's brow (Figure 1). At this distance, the visual speech stimuli filled the center 6° of visual space (±3° from midline). Auditory stimuli were paired with three possible types of videos, resulting in three conditions: Still Face, auditory stimuli paired with a motionless face; Congruent, auditory stimuli paired with their phonetically congruent videos; and Incongruent, auditory stimuli paired with a phonetically Incongruent video to create McGurk interference.

The main experiment lasted 1 hr and was divided into two halves. In each half, subjects were presented with stimuli from one of two sets; Set A (auditory /aba/ and /aga/) or Set B (auditory /apa/ and /aka/). Stimuli were presented in the Still Face, Congruent, and Incongruent (auditory /aba/ or /aga/ paired with visual /aga/ or /aba/ for Set A and auditory /apa/ or /aka/ paired with visual /aka/ or /apa/ for Set B) conditions. Each half consisted of three, 7 min sessions; each session consisted of 156 trials, for a total of 6 trials at each location, VCV, and condition. Locations, conditions, and VCVs were presented in pseudorandom order so that no single VCV, location, or condition was presented on more than three consecutive trials. Individual trials lasted approximately 2.6 s and consisted of three segments (Figure 1A). The first was a 500 ms fixation period following the loading of the first frame of the video at trial onset, designed to minimize potential transient attentional artifacts. This buffer was followed by a 1.0 s stimulus presentation period, which was in turn followed by an approximately 1.0 s response window. Subjects completed three sessions comprised of stimuli from either Set A or B before beginning the second set. Set order was counterbalanced across subjects.

Subjects were instructed to respond to two separate questions for each trial. First, subjects reported if they perceived the sound to originate from the speaker's mouth (S*ame-Location*) or somewhere else (*Different-Location*) by pressing their left index and middle fingers, respectively. Importantly, the experimenter repeatedly emphasized that the task was to compare the locations of what they heard and saw and that the presence or absence of mouth movement or whether or not the mouth movements matched what was heard was not part of the task. This emphasis helped ensure that any effects of condition are likely to be conservative. Additionally, subjects were instructed to maintain fixation throughout the experiment. The experimenter monitored for gross eye movements and any head movement via a remote camera placed in the testing room. Second, subjects reported which VCV they heard by pressing one of four buttons with their right index, middle, ring, and little fingers. These buttons were mapped to /aba/ or /aka/, /ada/ or /apa/, /aga/ or /ata/, and /other/ for Set A and Set B respectively. Although not explicitly presented, /ada/ and /ata/ were included as response options because subjects commonly report these percepts for auditory /b/ and /p/ paired with visual /g/ and /k/, respectively [16]. The /other/ category was included to allow for any unexpected response types. Subjects were instructed to press the /othe*r*/ button only if they could not unambiguously classify what they heard as one of the other three options. For each subject, speech sounds corresponding to the /other/ classification were noted by the experimenter during a short debriefing session.

Prior to beginning either set of stimuli, subjects completed a training session to learn the response mapping and task. This typically took 6–9 min and never exceeded 12 min of training. During these training sessions, each VCV was presented from −30°, 0°, or +30° degrees in the Still Face, Congruent, and Incongruent conditions. Subjects advanced to the main experiment once they responded *Same-Location* for a majority of sounds presented at 0° and *Different-Location* for most sounds presented at −30° or +30°. Prior to the start of each session, the experimenter placed a still image of the speaker's mouth in the center of the screen and looped a single auditory VCV located at 0°. The subject's head was maneuvered until the subject perceived the sound to originate from the *Same*-*Location* as the video. Care was taken to align the stimuli in both azimuth and elevation and yielded robust alignments (see Results). Stimulus presentation and response acquisition was coordinated with Neurobehavioral Systems' Presentation Software (www.neurobs.com).

### Corrected McGurk Interference Rate

Despite numerous studies employing McGurk interference, there is no clear consensus on how best to quantify McGurk interference rates. Here we report a “Corrected” McGurk interference rate that controls for highly confusable auditory signals in the absence of visual information. Specifically, the percentage of responses classified as McGurk Interference (e.g. /ada/ or /other/ responses for B/G audiovisual pairs and /ata/ or /other/ for K/P audiovisual pairs) in the Still Face condition was subtracted from the percentage of these responses in the Incongruent (McGurk) condition. That is, we quantified how much more likely a response category indicative of McGurk interference is when auditory stimuli are paired with visual Incongruent versus Still Face videos. Since VCV identities were generally more confused in the Still Face condition than in the Congruent condition (i.e. errors in reported VCV identity were generally higher in the Still Face condition, see Table 1), this measure resulted in a conservative estimate of McGurk interference and prevented artificially inflated McGurk interference rates due to ambiguous auditory stimuli.

**Table 1:** Confusion matrix for VCV identification. (% of Responses; mean ± SEM).

		Stimulus Condition		
Auditory Consonant	Response	Still Face	Congruent	Incongruent
B	B/P	75.9±4.5	90.6±4.4	10.8±7.9
	D/T	22.5±4.5	7.1±3.9	85.9±8.5
	G/K	1.1±0.3	1.3±0.5	1.9±0.5
	Other	0.5±0.3	1.0±0.4	1.3±0.6
G	B/P	3.2±0.9	1.7±0.5	2.0±0.9
	D/T	2.3±0.9	2.5±0.8	1.3±0.5
	G/K	84.3±4.8	91.2±2.5	35.0±11.0
	Other	10.1±3.8	4.6±1.8	60.9±11.1
P	B/P	79.1±5.0	89.2±2.9	15.6±8.6
	D/T	13.0±4.8	2.1±1.5	78.4±9.0
	G/K	5.4±1.3	5.6±1.3	2.8±1.4
	Other	1.7±1.5	2.3±1.8	2.4±1.6
K	B/P	2.8±1.0	2.7±0.8	3.2±1.1
	D/T	0.2±0.2	0.5±0.3	0.6±0.2
	G/K	92.2±2.7	95.2±1.5	40.9±9.7
	Other	2.7±1.5	1.9±1.1	53.8±10.2

### Psychometric Analysis

Psychometric analysis was performed by fitting the *%Same-Location* vs. Spatial-Disparity (Spatial-Location collapsed across side) for each subject and condition with a sigmoid curve [Y = 1/(1 + exp(-A*(X-B)))] with MATLAB's ‘fit’ function. Parameter A is indicative of the slope of the function while B is the threshold while X and Y represent the independent (here, spatial disparity (°)) and dependent (% *Same-Location*) variables, respectively. Parameter estimates were included in separate one-factor ANOVAs to assess any main effect of stimulus Condition [21].

### Statistical Methods

All statistical tests were performed in STATISTICA version 8.0 (www.statsoft.com). All reported p-values have been Greenhouse-Geisser corrected for non-sphericity. Post-hoc tests were performed using Fisher's least significant difference (LSD). Unless otherwise noted, results are reported as mean ± standard error of the mean (SEM).

## Results

### HRTF validation and head alignment

**Figure 2:**
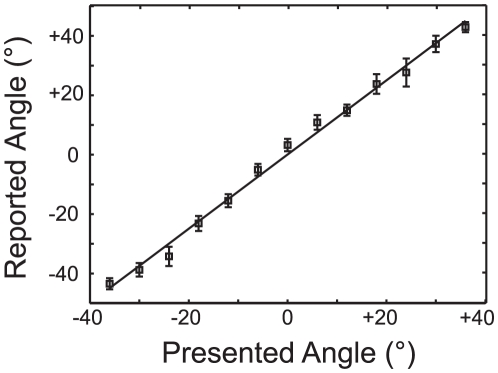
HRTFs precisely capture binaural auditory cues. Subjects indicated the perceived location of an HRTF filtered speech token (e.g. /aba/) at one of thirteen different locations via a pointing task. These indicated locations are plotted as a function of the intended (experimenter controlled) location. The data show a linear relationship between the Presented Angle and Reported Angle (1.25*X+0.176, R^2^ = 0.99). (mean ± SEM, N = 10).

HRTFs are notoriously difficult to estimate well and are often plagued by acoustic artifacts [20]. As a result, we conducted a validation experiment with ten individuals (five of whom participated in the main experiment; see Methods for details) to demonstrate that our HRTF protocol captures cues important in azimuthal sound. Figure 2 plots reported angles as a function of presented angle. Responses were remarkably precise both within and between subjects and were well explained by a linear polynomial (y = 1.25*X+0.176, R^2^ = 0.99). Thus, our HRTF estimation routine provides a precise (although with slightly overestimated slope by this measure) representation of acoustic space for all subjects.

An additional challenge to using HRTFs in the current study is that slight changes in head position result in audiovisual stimuli falling out of spatial registry. For instance, a slight rotation of the midsaggital plane to the right will result in auditory space shifting to the right while visual stimuli remain anchored in space. To verify that our efforts to align auditory and visual space were robust across subjects (see Methods), we fit the % *Same-Location* vs. Location function (collapsed across conditions) with a Gaussian function in MATLAB for each subject. The means of these Gaussian distributions were then subjected to a t-test. A mean of 0° would indicate perfect alignment while any non-zero value would indicate a systematic misalignment. Although individual subject peaks occasionally deviated from 0° (maximum of 6°, median 0.135°), there was no systematic relationship in the direction or magnitude of this shift (p = 0.58; mean = −0.07±1.21° degrees). Together, these data suggest that our protocol captures cues important in sound localization and ensures robust audiovisual alignment.

### Speech Classification and McGurk Interference

On average, subjects were able to correctly identify auditory VCVs on 83.50±3.07% of trials in the Still Face condition (see Table 1). Identification performance improved to 91.52±2.34% in the Congruent condition (p<0.001; mean difference [d-] = 8.02±1.74%), suggesting that subjects used the visual information to aid in VCV identification. Ten of the eleven subjects experienced McGurk interference and tended to respond /ada/ for B/G, /ata/ for P/K, /other/ (typically /abga/) for G/B, and /other/ (typically /apka/) for K/P audiovisual pairs (see Table 1). The remaining subject did not reliably experience McGurk interference with any of the four McGurk pairs but was still included in the analysis.

**Figure 3:**
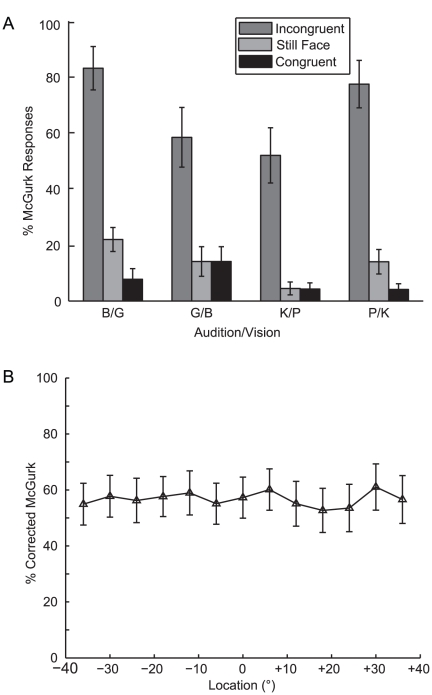
McGurk interference is spatially independent. (A) McGurk interference rates are plotted as a function of stimulus condition and audiovisual pair. Responses indicative of McGurk interference significantly increased in the Incongruent condition compared to the Still Face and Congruent conditions. (B) Corrected McGurk interference rates are plotted as a function of location. The data suggest that speech related cues operate independently of spatial information. Corrected McGurk interference rates were calculated by subtracting the %McGurk Responses in the Still Face Condition (light-gray bars) from the Incongruent condition (dark-gray bars). (N = 11, means ± SEM).

The (uncorrected) percentage of McGurk type responses is plotted as a function of condition and audiovisual pair in Figure 3A and Corrected McGurk Interference rates are plotted as a function of location in Figure 3B. Corrected McGurk Interference rates were collapsed across side and included in a two-factor [Spatial-Disparity x VCV] repeated-measure ANOVA. The main effect of VCV was insignificant (p = 0.23; η^2^_p_ = 0.14) and did not interact with Spatial-Disparity (p = 0.22; η^2^_p_ = 0.12). Finally, the main effect of Spatial-Disparity was insignificant (p = 0.54; η^2^_p_ = 0.07; 60.02±7.38% for 0°, 59.23±7.22% for 6°, 58.15±7.53 for 12°, 56.84±7.24% for 18°; 56.68±7.97% for 24°; 60.86±7.39% for 30°; 56.87±7.63% for 36°). Together, these data suggest that McGurk interference rates were consistent across audiovisual pairs and, more importantly, that speech cues are integrated independently of audiovisual spatial attributes, as suggested by previous studies (e.g. [17], [22], [23], but see [24]).

### Conflicting Speech Cues Inhibit Spatial Integration

**Figure 4:**
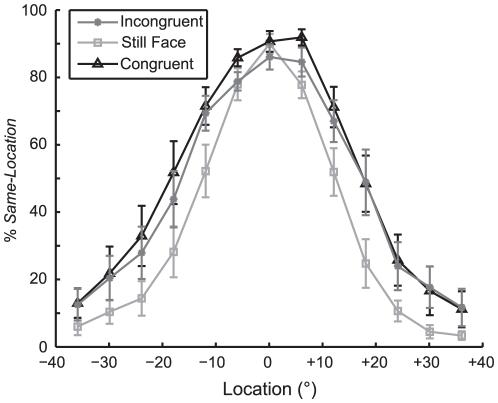
Speech cues affect audiovisual integration in space. (A) The percentage of *Same-Location* responses is plotted as a function of location for each condition: Congruent (black triangles), Incongruent (dark gray, asterisk), and Still Face (light gray, squares). Importantly, the likelihood of spatial integration (i.e. responding *Same-Location*) decreases at the smallest disparities in the Incongruent relative to the Congruent condition. (N = 11, means ± SEM).

In contrast to the spatial insensitivity of McGurk interference, the ventriloquist's illusion is highly dependent on audiovisual spatial attributes (Figure 4). To determine the contribution of higher-order speech related cues to audiovisual spatial integration, we measured the percentage *Same-Location* responses with sounds paired with their phonetically Congruent or Incongruent videos. Critically, both Congruent and Incongruent stimuli maintained their temporal registry to within the temporal precision of the video (see Methods for more details). Thus any differences observed between conditions are unlikely due to differential temporal alignment.

The percentage of *Same-Location* responses is plotted as a function of location in Figure 4. As audiovisual spatial disparity increased in either direction, the likelihood of a *Same-Location* response decreased monotonically, resulting in a smooth function symmetric about 0°. Despite spatial precision on the order of 3-5° during a spatial pointing task (see Figure 2), audiovisual spatial comparisons tended to be less precise in the S*ame-* or *Different-Location* task. For instance, in the Still Face condition (light grey squares) subjects responded *Same-Location* on approximately 25% of trials with an 18° spatial disparity. In contrast, subjects participating in the pointing task never confused a sound presented at 18° with the 0° location. Despite this nuance, subjects were more likely to respond *Same-Location* in the Congruent condition than in the Still Face condition (p<0.001; 50.95±3.74% for Congruent and 37.95±2.52% for Still Face; d- = 13.00±2.23%). We interpret this increase in the percentage of *Same-Location* responses as the ventriloquist's illusion [22].

To determine whether phonetic cues contribute to audiovisual integration in space, we included the percentage of *Same-Location* responses, collapsed across side, in a three-factor [Spatial-Disparity x Condition x VCV] repeated-measure ANOVA. The main effects of Spatial-Disparity (p<0.001; η^2^_p_ = 0.90; 88.18±2.68% for 0°, 82.08±2.29% for 6°, 63.10±4.58% for 12°, 40.24±7.00% for 18°, 21.87±5.22% for 24°, 14.42±3.97% for 30°, and 8.86±2.81% for 36°; 0°,6°>12°>18°>24°,30°>36°; pairwise comparisons with Fisher's Least Significant Difference [LSD]) and Condition (p<0.001; η^2^_p_ = 0.70; 50.95±3.74% for Congruent, 47.70±3.41% for Incongruent, 37.95±2.52% for Still Face; Congruent, Incongruent > Still Face) were significant while the main effect of VCV fell below significance after Greenhouse-Geisser correction (p<0.05). More importantly, VCV did not interact with any other factor with or without Greenhouse-Geisser correction (p>0.13), suggesting that all speech tokens were spatially equivalent. Instead, only Spatial-Disparity and Condition significantly interacted (p<0.006; η^2^_p_ = 0.34). Post-hoc tests revealed a significantly greater likelihood of responding *Same-Location* in the Congruent condition relative to the Still Face condition at all non-zero spatial disparities. In contrast, the percentage of S*ame-Location* responses was significantly different between Incongruent and Still Face conditions at all audiovisual disparities except 6° (p = 0.20) and 0° (p = 0.18). Most importantly, there was a significant difference between Congruent and Incongruent conditions at the smallest measured disparity of 6°. Specifically, the percentage of *Same-Location* responses decreased by 11.03±3.94%, virtually abolishing the ventriloquist's illusion (p = 0.016). Thus, conflicting speech related cues significantly attenuated audiovisual integration in space, but only at the smallest measured disparity.

There are several mechanisms through which the ventriloquist's illusion can manifest in its own right and be modified by conflicting phonetic cues: by 1) decreasing auditory spatial sensitivity (i.e. a change in the slope of a psychometric function), 2) shifting the dynamic range (i.e. a change in threshold of a psychometric function), or 3) both a change in dynamic range and spatial sensitivity. To further clarify how these potential mechanisms contribute to audiovisual spatial integration, we compared slope and threshold parameter estimates of a logistic curve fit to each subject's %*Same-Location* vs. Spatial-Disparity function for each condition (see Methods for details). The slope parameter was −0.22±0.03 in the Still Face, −0.20±0.4 in the Congruent, and −0.19±0.04 in the Incongruent conditions. A one-factor, repeated-measure ANOVA revealed no significant difference between conditions (p = 0.19; η^2^ = 0.158). In contrast, psychometric thresholds (Spatial-Disparity yielding 50% *Same-Location* responses) differed between conditions (p<0.001; η^2^ = 0.706; 12.67±1.02° for Still Face, 19.12±1.77° for Congruent, and 17.73±1.70° for Incongruent conditions). Post-hoc tests revealed a significant difference between the Still Face and Congruent (p = <0.001; η^2^ = 0.78; d- = 6.45±1.09°) and Still Face and Incongruent (p = <0.001; η^2^ = 0.73; d- = 5.05±0.98°) conditions, but no difference between Congruent and Incongruent conditions (p = 0.17; η^2^ = 0.21; d- = 1.40±0.86°).

## Discussion

The brain uses information from multiple modalities to construct a statistically optimal representation of our world. Particularly important for human communication and the focus of this study is audiovisual integration. Our results provide new evidence about these integrative mechanisms by suggesting that higher order speech cues can guide audiovisual spatial integration, while the converse is not necessarily true. Specifically, although integrative processing of speech related cues operates independently of spatial information (e.g. McGurk interference), conflicting speech cues can affect the likelihood of spatial integration when their lower-level information might erroneously drive integration. These observations may be a direct consequence of asymmetrical information sharing between processing streams in the brain. For instance, existing evidence suggests that both visual and auditory information is processed along dorsal ‘where’ and ventral ‘what’ pathways involved in processing object location and identity, respectively [25], [26], [27]. Our data suggest that information sharing between these pathways may be asymmetric.

McGurk interference, a classic instance of audiovisual integration, served as a key element of our design. In agreement with prior reports [17], [22], [23], [28] (but see [24]), we show that the McGurk illusion depends on conflicting speech-related cues (here, place of articulation) but is utterly independent of spatial disparity. This suggests that processing of speech-cues, likely along the ventral ‘what’ pathway, does not necessarily have access to auditory or visual spatial information. We would not, however, argue that such object-related integrative processes are strictly automatic or isolated from ongoing cognition and perception. In fact, recent evidence suggests that top-down cognitive factors, such as attention, can affect the likelihood of integrating audiovisual objects. For instance, McGurk interference can break down when subjects are engaged in an attentionally demanding task [29], [30]. Together, this evidence shows that while audiovisual integration of high-level cues may benefit from supramodal cognitive resources, it proceeds without regard for spatial representations. Interestingly, as discussed below, the converse is not true: high-level cues can in fact affect spatial integration.

Our results show that speech cues are relevant for audiovisual spatial integration. Specifically, when audiovisual speech with virtually identical spatiotemporal properties is manipulated to create phonetically Congruent and Incongruent (McGurk) audiovisual pairs, visual spatial capture (i.e. the ventriloquist's illusion) is consistently attenuated in the Incongruent condition relative to its Congruent counterpart. Interestingly, this inhibitory effect only occurs when relatively low-level audiovisual spatial information nearly coincides. These data suggest that spatial processing, likely along the dorsal ‘where’ pathway, has access to higher order information from the ventral ‘what’ pathway. Although the neural mechanism remains elusive, perhaps the ventral stream encodes an error signal that inhibits spatial integration when object identities fail to match, but is only compelling enough to override highly confusable signals.

Importantly, the data do not suggest that conflicting phonetic cues cause global changes in auditory spatial processing, as a more detailed psychometric analysis of the Congruent and Incongruent conditions revealed no consistent changes in auditory spatial sensitivity (slope of the function) or the dynamic range of our subjects' auditory spatial sensitivity. Instead, this behaviorally consequential phenomenon seems only to prevent signals that are highly confusable according to spatiotemporal proximity, as described originally by Stein and Meredith [12], from being erroneously integrated.

Together our data combined with accumulating evidence from recent studies [29], [30] suggest that multisensory integration is not governed solely by low-level spatial and temporal properties of the stimuli, as might be suggested by early neural models of integration [12]. Instead, we argue that the brain's effort to integrate information of common origin is often influenced by additional, abstract stimulus properties, such as speech cues and ongoing cognitive demands, such as attention [29], [30]. Future studies might further explore the relationship between these newly appreciated contributors under more realistic conditions. Perhaps, in contrast to the current findings, spatial cues contribute to the integration of speech cues but under more challenging circumstances, for instance with multiple competing talkers. To our knowledge, such competitive audiovisual speech tasks are rare (see [31] for a recent example) and have so far not exploited McGurk interference to dissect the underlying neural mechanisms. This could be due in part to the inherent difficulty in exploring McGurk interference with peripheral visual stimuli, a likely necessity in such experiments. Specifically, McGurk interference is known to diminish rapidly as visual stimuli move from foveal to peripheral locations [28], probably due to spatial smoothing of the stimuli in the visual periphery [32]. Although these experiments may prove difficult, they would undoubtedly provide a deeper understanding of complex, real-world multisensory integration.

In contrast to our findings, a recent report by Colin et al. (2001) found that spatial integration operates independently of phonetic identity [22]. However, this apparent contradiction is easily reconciled when one considers the details of both experiments. Colin et al. recorded the likelihood of responding S*ame-Location* from ±80° in 20° increments. In contrast, we recorded responses at a much higher spatial resolution, and, more importantly, at disparities smaller than 20°. In the current report, we also fail to find differences in audiovisual spatial integration between Congruent and Incongruent conditions at disparities larger than 6° and would have arrived at identical conclusions had we not used such high spatial sampling. Once these factors are taken into account, the two independent studies corroborate one another despite reaching qualitatively different conclusions.

In sum, audiovisual integration is vital for day-to-day navigation and communication and is strongly driven by bottom-up spatial and temporal evidence. However, we demonstrate here that some integrative phenomena, such as McGurk interference, operate independently of spatial processing. In contrast, conflicting speech cues can impact audiovisual integration in space. These data suggest that under some circumstances, information is shared asymmetrically between dorsal ‘where’ and ventral ‘what’ processing streams. Future studies might explore how other high-level cognitive and perceptual properties affect this balance of influence during audiovisual integration of speech in noisy environments.

## References

[1] Ernst MO, Bulthoff HH (2004). Merging the senses into a robust percept.. Trends Cogn Sci.

[2] Shahin AJ, Miller LM (2009). Multisensory integration enhances phonemic restoration.. J Acoust Soc Am.

[3] Sumby WH, Pollack I (1954). Visual Contribution to Speech Intelligibility in Noise.. Journal of the Acoustical Society of America.

[4] Bishop CW, Miller LM (2009). A Multisensory Cortical Network for Understanding Speech in Noise.. J Cogn Neurosci.

[5] Ernst MO, Banks MS (2002). Humans integrate visual and haptic information in a statistically optimal fashion.. Nature.

[6] Cherry EC (1953). Some Epxeriments on the Recognition of Speech, with One and with Two Ears.. J Acoust Soc Am.

[7] Bizley JK, King AJ (2009). Visual influences on ferret auditory cortex.. Hearing Research.

[8] Alais D, Burr D (2004). The ventriloquist effect results from near-optimal bimodal integration.. Current Biology.

[9] Bizley JK, King AJ (2008). Visual-auditory spatial processing in auditory cortical neurons.. Brain Res.

[10] Bizley JK, Nodal FR, Bajo VM, Nelken I, King AJ (2007). Physiological and anatomical evidence for multisensory interactions in auditory cortex.. Cereb Cortex.

[11] Meredith MA, Nemitz JW, Stein BE (1987). Determinants of multisensory integration in superior colliculus neurons. I. Temporal factors.. J Neurosci.

[12] Meredith MA, Stein BE (1983). Interactions among converging sensory inputs in the superior colliculus.. Science.

[13] Meredith MA, Stein BE (1996). Spatial determinants of multisensory integration in cat superior colliculus neurons.. J Neurophysiol.

[14] Meredith MA, Wallace MT, Stein BE (1992). Visual, auditory and somatosensory convergence in output neurons of the cat superior colliculus: multisensory properties of the tecto-reticulo-spinal projection.. Exp Brain Res.

[15] Slutsky DA, Recanzone GH (2001). Temporal and spatial dependency of the ventriloquism effect.. Neuroreport.

[16] McGurk H, MacDonald J (1976). Hearing lips and seeing voices.. Nature.

[17] Jones JA, Munhall K (1997). The effects of separating auditory and visual sources on audovisual integration of speech.. Canadian Acoustics.

[18] Howard IP, Templeton WB (1966). Human Spatial Orientation..

[19] Warren DH, Welch RB, McCarthy TJ (1981). The role of visual-auditory "compellingness" in the ventriloquism effect: implications for transitivity among the spatial senses.. Percept Psychophys.

[20] Blauert J (1996). Spatial Hearing - Revised Edition: The Psychophysics of Human Sound Localization..

[21] Recanzone GH (2003). Auditory influences on visual temporal rate perception.. J Neurophysiol.

[22] Colin C, Radeau M, Deltenre P, Morais J (2001). Rules of intersensory integration in spatial scene analysis and speechreading.. Psychologica Belgica.

[23] Bertelson P, Vroomen J, Wiegeraard G, De Gelder B (1994). Exploring the relation between McGurk Interference and Ventriloquism..

[24] Jones JA, Jarick M (2006). Multisensory integration of speech signals: the relationship between space and time.. Exp Brain Res.

[25] Belin P, Zatorre RJ (2000). ‘What’, ‘where’ and ‘how’ in auditory cortex.. Nat Neurosci.

[26] Mishkin M, Ungerleider LG, Macko KA (1983). Object vision and spatial vision: two cortical pathways.. Trends Neurosci.

[27] Romanski LM, Tian B, Fritz J, Mishkin M, Goldman-Rakic PS (1999). Dual streams of auditory afferents target multiple domains in the primate prefrontal cortex.. Nat Neurosci.

[28] Pare M, Richler RC, ten Hove M, Munhall KG (2003). Gaze behavior in audiovisual speech perception: the influence of ocular fixations on the McGurk effect.. Percept Psychophys.

[29] Alsius A, Navarra J, Campbell R, Soto-Faraco S (2005). Audiovisual integration of speech falters under high attention demands.. Curr Biol.

[30] Alsius A, Navarra J, Soto-Faraco S (2007). Attention to touch weakens audiovisual speech integration.. Exp Brain Res.

[31] Senkowski D, Saint-Amour D, Gruber T, Foxe JJ (2008). Look who's talking: the deployment of visuo-spatial attention during multisensory speech processing under noisy environmental conditions.. Neuroimage.

[32] MacDonald J, Andersen S, Bachmann T (2000). Hearing by eye: how much spatial degradation can be tolerated?. Perception.

